# MEG Centrality scores as indicators of inhibitory neuron dysfunction in healthy individuals at risk of AD and early effects of white matter hyperintensities on MEG

**DOI:** 10.1002/alz70856_097704

**Published:** 2025-12-24

**Authors:** Alejandra García‐Colomo

**Affiliations:** ^1^ Centro de Neurociencia Cognitiva y Computacional, Madrid, Spain; Center for Cognitive and Computational Neuroscience, Madrid, Madrid, Spain; Universidad Complutense de Madrid, Madrid, Madrid, Spain

## Abstract

**Background:**

The definition of Alzheimer's disease (AD) as a biological continuum enabled the identification of distinct stages preceding dementia. This framework highlights the importance of early identification, which could facilitate the implementation of disease‐modifying interventions. Consequently, the search for early biomarkers began. Electrophysiological measures have proven highly relevant, as they detect functional alterations from the earliest stages of incipient pathology throughout the entire continuum. In this talk, we will discuss the electrophysiological alterations associated with different markers of pathology, including plasma *p*‐tau231 (i.e. an Aβ proxy), NfL and white matter hyperintensities among cognitively unimpaired (CU) individuals and mild cognitive impairment (MCI) patients.

**Method:**

404 CU individuals and 117 MCI patients underwent a magnetoencephalography (MEG) resting‐state scan. For 104 CU individuals, plasma *p*‐tau231 and NfL levels were available, and their association with the compound centrality score of each brain source was evaluated. The remaining 300 CU individuals and the MCI group had available data on white matter hyperintensities. Their relationship with spectral power was assessed.

**Result:**

Increasing *p*‐tau231 concentrations in CU individuals were associated with greater theta centrality in posterior areas, and lower gamma centrality in left regions. Hubs were most vulnerable to the theta centrality shift. CU and MCI individuals with vascular damage showed beta relative power reductions that were associated with structural integrity.

**Conclusion:**

The observed centrality alterations align with the early hub vulnerability associated with Aβ deposition and the resulting inhibitory neuronal dysfunction. Meanwhile, the spectral signatures of vascular impairment are in line with previous literature, but greater consensus on the definition of this clinical entity and more replication studies are paramount.

